# Discovering urinary bladder cancer risk variants: Status quo after almost ten years of genome-wide association studies

**DOI:** 10.17179/excli2017-1000

**Published:** 2017-12-08

**Authors:** Silvia Selinski

**Affiliations:** 1 Leibniz Research Centre for Working Environment and Human Factors (IfADo)

## ⁯

About ten years ago Kiemeney and colleagues (2008[24]) published the first genome-wide association study (GWAS) discovering two novel single nucleotide polymorphisms (SNPs) near *MYC* (rs9642880) and *TP63* (rs710521) associated with urinary bladder cancer (UBC) risk. Meanwhile, further GWAS and candidate gene studies identified and confirmed a number of susceptibility variants for UBC (Selinski, 2012[37], 2013[38], 2014[39]; Dudek et al., 2013[4]; Selinski et al., 2013[41]; Golka et al., 2011[18]). Currently, fifteen genomic regions seem to play a major role in development of this disease (Table 1(Tab. 1); References in Table 1: Figueroa et al., 2014[8]; Figueroa et al., 2016[7]; Fu et al., 2012[9]; Garcia-Closas et al., 2011[12]; Garcia-Closas et al., 2013[11]; Golka et al., 2009[13]; Grotenhuis et al., 2014[20]; Kiemeney et al., 2008[24]; Kiemeney et al., 2010[23]; Lehmann et al., 2010[26]; Nørskov et al., 2011[29]; Rafnar et al., 2009[31]; Rafnar et al., 2011[33]; Rafnar et al., 2014[32]; Rothman et al., 2010[34]; Selinski et al., 2011[42]; Selinski et al., 2012[44]; Tang et al., 2012[45]; Wang et al., 2014[46]; Wu et al., 2009[47]). It can be assumed that almost all relevant polymorphisms have been discovered now. The most recent UBC GWAS of Figueroa et al. (2016[7]) required 15,058 cases and 286,270 controls to discover a further susceptibility region at 13q34 (*MCF2L* gene), including the Icelandic and the Dutch GWAS (Gudbjartsson et al., 2015[21]; Kiemeney et al., 2008[24]). The odds ratios of the most recent UBC risk SNPs are rather small, e.g. the *MCF2L* intron variant rs4907479, with the strongest signal in the 2016 fine-mapping study of Figueroa et al. (2016[7]) resulted in an odds ratio (OR) of 1.13. 

Several UBC risk variants are particularly relevant in persons exposed to bladder carcinogens - mainly by tobacco smoke (Burger et al., 2013[1]; Garcia-Closas et al., 2005[10], 2011[12], 2013[11]; Selinski et al., 2013[41]; Moore et al., 2011[28]) but also by occupation and environment (Ebbinghaus et al., 2017[5]; Höhne et al., 2017[22]; Krech et al., 2017[25]; Lukas et al., 2017[27]; Carreón et al., 2014[2]; Golka et al., 2012[14], 2009[13], 2004[19], 2002[15], 1997[17], 1996[16]; Delclos and Lerner, 2008[3]; Ovsiannikov et al., 2012[30]; Rushton et al., 2012[35]). However, currently no particular variant that is only relevant in exposed persons could be identified and replicated in GWAS. In 2014, Figueroa et al. (2014[6]) identified two variants in a genome-wide smoking ⨯ SNP interaction study -rs1711973 (*FOXF2*) relevant for never smokers and rs12216499 (*RSPH2*-*TAGAP*-*EZR*) in ever smokers, but both could not be confirmed using a large replication series (Figueroa et al., 2016[7]). Nevertheless, interaction analyses and stratification regarding smoking habits and cancer invasiveness are promising future approaches to uncover further susceptibility variants that are relevant for particular subgroups of the UBC patients. 

A further challenge is a genome-wide search for variants associated with bladder cancer recurrence and progression. This requires a large number of UBC patients with follow-up of several years after first diagnosis. However, it can be assumed that variants relevant for UBC development are also associated with UBC recurrence. Recurrence of this tumor occurs in approximately half of the patients with a median recurrence-free time of almost one year. Selinski et al. (2017[43]) showed that the ultra-slow N-acetyltransferase 2 (*NAT2*) genotype was associated with a significant reduction of recurrence-free time (8.4 months) compared to rapid acetylators (11 months) and an increased recurrence risk (66 % vs. 50 %, OR=1.89, 95 % CI = 1.06-3.38). Effects were more pronounced in ultra-slow smokers (7.9 months, 73 %) indicating the relevance of gene-environment interaction also for prognosis. Correspondingly, Lukas et al. (2017[27]) showed in a series of 143 UBC cases with suspected occupational bladder cancer the importance of co-occurring susceptibility variants, particularly co-occurring *GSTM1* negative and rs11892031[A/A] for UBC recurrence. They discovered that UBC cases with an elevated number of risk alleles had a significantly shorter median relapse-free time of 8 months compared to cases with few risk alleles. 

According to the different importance of several genetic variants depending on exposure to bladder carcinogens, e.g. *GSTM1* and *NAT2*, different variant combinations seem to play a major role in smokers and never smokers (Selinski et al., 2017[40]; Schwender et al., 2012[36]. Recently, Selinski et al. (2017[40]) identified and replicated in a large multi-centric case-controls series (discovery series: 2969 cases / 3285 controls, replication series: 2080 cases / 2167 controls) four-variant combinations out of twelve well-known UBC risk variants. The highest odds ratios were found in never smokers with the best combination (rs1014971[AA] ⨯ rs1058396[AG,GG] ⨯ rs11892031[AA] ⨯ rs8102137[CC,CT]) resulting in an OR of 2.59 (95 % CI = 1.93-3.47; P = 1.87 ⨯ 10^-10^, frequency in never smoking cases: 25 %). Odds ratios of the best combinations found in smokers were clearly lower (current smokers: 1.56, former: 2.13, ever: 1.55) and different variant combinations were relevant, especially *GSTM1*, rs1058396 (*SLC14A1*) and rs11892031 (*UGT1A*) combinations in current and rs9642880 (*MYC*), rs1495741 (*NAT2*) and rs8102137 (*CCNE1*) combinations in former smokers (Selinski et al., 2017[40]). 

## Figures and Tables

**Table 1:**
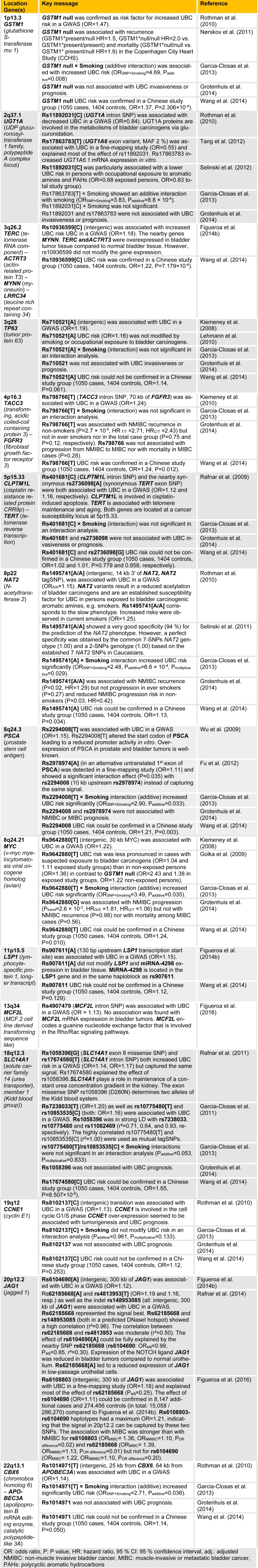
Currently confirmed polymorphisms that are associated with UBC risk, their association with bladder carcinogen exposure and prognosis (update of Selinski, 2014) according to Selinski (2014). Polymorphisms, associated genes and locations are printed in bold, risk alleles or genotypes are given in brackets.
